# The role of *Candida albicans *homologous recombination factors Rad54 and Rdh54 in DNA damage sensitivity

**DOI:** 10.1186/1471-2180-11-214

**Published:** 2011-09-27

**Authors:** Samantha J Hoot, Xiuzhong Zheng, Catherine J Potenski, Theodore C White, Hannah L Klein

**Affiliations:** 1 Department of Biochemistry, New York University School of Medicine, 550 First Avenue, New York, NY 10016, USA; 2 Seattle Biomedical Research Institute, Seattle, WA 98109, USA; 3 School of Biological Sciences, University of Missouri-Kansas City, 5007 Rockhill Road, Kansas City, MO 64110, USA

## Abstract

**Background:**

The fungal pathogen *Candida albicans *is frequently seen in immune suppressed patients, and resistance to one of the most widely used antifungals, fluconazole (FLC), can evolve rapidly. In recent years it has become clear that plasticity of the *Candida albicans *genome contributes to drug resistance through loss of heterozygosity (LOH) at resistance genes and gross chromosomal rearrangements that amplify gene copy number of resistance associated genes. This study addresses the role of the homologous recombination factors Rad54 and Rdh54 in cell growth, DNA damage and FLC resistance in *Candida albicans*.

**Results:**

The data presented here support a role for homologous recombination in cell growth and DNA damage sensitivity, as *Candida albicans rad54Δ/rad54Δ *mutants were hypersensitive to MMS and menadione, and had an aberrant cell and nuclear morphology. The *Candida albicans rad54Δ/rad54Δ *mutant was defective in invasion of Spider agar, presumably due to the altered cellular morphology. In contrast, mutation of the related gene *RDH54 *did not contribute significantly to DNA damage resistance and cell growth, and deletion of either *Candida albicans RAD54 *or *Candida albicans RDH54 *did not alter FLC susceptibility.

**Conclusions:**

Together, these results support a role for homologous recombination in genome stability under nondamaging conditions. The nuclear morphology defects in the *rad54Δ/rad54Δ *mutants show that Rad54 performs an essential role during mitotic growth and that in its absence, cells arrest in G2. The viability of the single mutant *rad54Δ/rad54Δ *and the inability to construct the double mutant *rad54Δ/rad54Δ rdh54Δ/rdh54Δ *suggests that Rdh54 can partially compensate for Rad54 during mitotic growth.

## Background

The pathogenic yeast *Candida albicans *is one of the most common causes of fungal infection in immune compromised patients. There is a limited spectrum of antifungal drugs to which *C. albicans *is susceptible, which includes the azoles, amphotericin B and the echinocandins. The azole drug fluconazole (FLC) is a commonly used drug to treat oropharyngeal candidiasis but resistance to this drug can develop rapidly in the clinical setting. FLC has long been used to treat cases of life-threatening invasive candidiasis, but the emergence of azole resistance has favored the use of the echinocandins in invasive disease [1].

Resistance to the azoles can develop through a number of mechanisms, including point mutations or overexpression of a number of resistance genes. Genes known to be involved in *Candida albicans *resistance to FLC include the drug efflux pumps encoded by *CDR1, CDR2 *and *MDR1*, the FLC target encoded by *ERG11*, (lanosterol 14-alpha-demethylase) and the transcription factors that control the expression of these genes [2-6]. Studies of FLC resistant clinical and laboratory derived isolates of *Candida albicans *have shown that point mutations followed by loss of heterozygosity (LOH) events can further increase resistance [7-9]. Recent work has shown that gross chromosomal rearrangements that lead to aneuploidy and isochromosome formation contribute to FLC resistance by amplification of *ERG11 *and *TAC1 *mutant alleles [10,11]. This evidence suggests that the plasticity of the *Candida albicans *genome provides a selective advantage in certain environmental conditions, such as exposure to antifungal drugs.

Work to elucidate the mechanism that leads to these types of genome events in *Candida albicans *has shown that certain DNA repair mechanisms are not involved. For example, mechanisms such as non-homologous end joining, base excision repair and nucleotide excision repair do not appear to contribute significantly to the development of FLC resistance [12,13]. However, there is some evidence suggesting a role for homologous recombination in FLC resistance, as deletion of *RAD50, RAD52 *or *MRE11 *in *Candida albicans *alters FLC susceptibility [12].

The role that homologous recombination plays in FLC susceptibility and genome plasticity is not fully understood, although it is known that homologous recombination pathways preserve genome structure. Since the LOH events seen in FLC resistance *Candida albicans *clinical isolates likely occurs through a recombination mediated event, homologous recombination pathways may be required for LOH in clinical isolates, and defects in homologous recombination may inhibit the development of FLC resistance through LOH events. To further understand the role that homologous recombination pathways play in genome maintenance and DNA damage resistance in *Candida albicans*, we have examined the phenotypes of two genes proposed to be involved in homologous recombination based on their homology to the *Saccharomyces cerevisiae *genes. In *Saccharomyces cerevisiae*, two members of the *SNF2 *family of chromatin remodelers, *RAD54 *and *RDH54 *act in the repair of double strand DNA breaks through homologous recombination [14-16]. In vitro data suggest that Rad54 and Rdh54 act at stages of recombination involving strand displacement and D-loop formation [17]. *RAD54 *and *RDH54 *belong to the *RAD52 *epistasis group, which contains genes required for repair of double strand breaks generated through spontaneous events or exogenous damage. In humans, two *RAD54 *homologues, hRAD54 and RAD54B are present, and mutation of these is associated with tumor formation [18-20]. Despite similar *in vitro *activities of the Rad54 and Rdh54 proteins, the *Saccharomyces cerevisiae *mutants have different phenotypes with respect to mitotic and meiotic recombination [16] and DNA damage [14].

The work presented here on *Candida albicans RAD54 *and *RDH54 *examines the role these genes play in DNA damage sensitivity and in FLC susceptibility in *Candida albicans*. We found that *Candida albicans RAD54 *is required for normal cell growth and in its absence cells had an aberrant cell cycle, misdivide the nucleus, and appeared to have a DNA damage checkpoint arrest. In contrast, we found no DNA damage sensitivity or alteration of the cell cycle in *rdh54Δ/rdh54Δ *mutants. We did not observe a changed growth response to FLC, but merely observed slower growth of the *rad54Δ/rad54Δ *strain with or without FLC. Interestingly, *Candida albicans RAD54 *and *RDH54 *appeared to have some functional overlap as we were unable to construct the double mutant *rad54Δ/rad54Δ rdh54Δ/rdh54Δ*.

## Results

### Identification of Candida albicans homologues of Saccharomyces cerevisiae RAD54 and RDH54

To identify putative homologues of *Saccharomyces cerevisiae RAD54 *and *RDH54*, the protein sequence from each ORF was used for BLAST analysis. For each protein, putative homologues encoded in the *Candida albicans *genome were identified. For Rad54, a BLAST score of 1.6e^-245 ^and 69% amino acid identity over the region of highest homology was obtained. BLAST analysis of Rdh54 identified a homologue with a score of 2.6e^-128 ^and with 45% amino acid identity. The genes identified in the BLAST searches correspond to ORF 19.5004 and 19.5367, respectively in the Candida Genome Database maintained at Stanford University (http://www.candidagenome.org). Expression profiling of *Candida albicans *throughout the cell cycle has indicated that *Candida albicans RAD54 *expression peaks in S-phase and *Candida albicans RDH54 *peaks approximately at the G1/S transition [21].

### Candida albicans RAD54 deletion results in a slow growth phenotype

**Figure 1:**
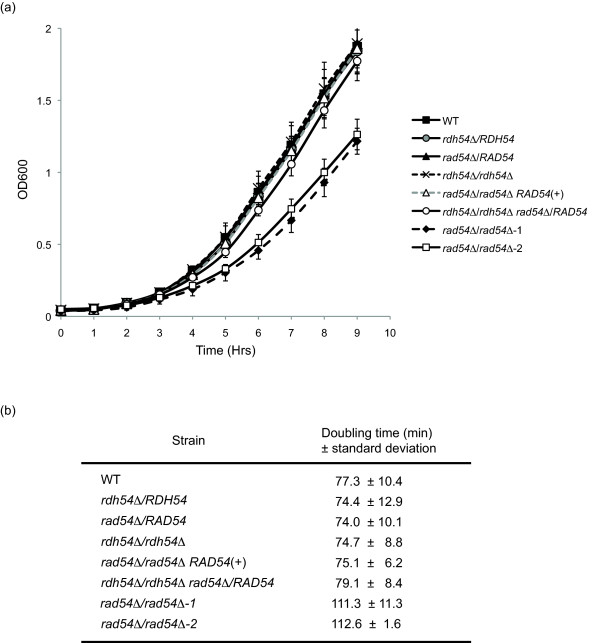
**Growth curves and doubling times of *rad54Δ/rad54Δ *and *rdh54Δ/rdh54Δ *strains**. A. Log phase growth curves for the indicated strains are shown. Two independent *rad54Δ/rad54Δ *strains were used, which are designated as 1 and 2. B. Doubling times for the indicated strains, derived from the data shown in panel A. Two independent *rad54Δ/rad54Δ *strains were used, which are designated as 1 and 2.

**Figure 2:**
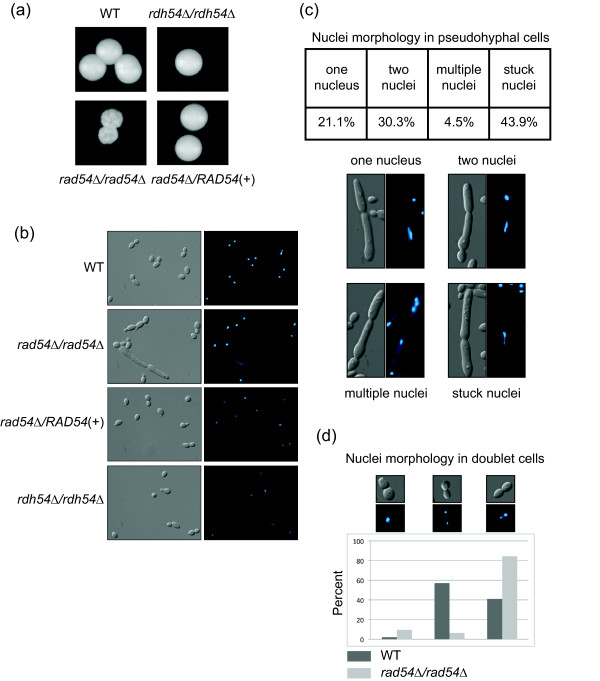
**Colony and cell morphology of *rad54Δ/rad54Δ *and *rdh54Δ/rdh54Δ *strains**. A. Colony morphology after three days of growth on YPD is shown. B. DIC images and DAPI images of strains of the indicated genotypes. Note the aberrant cell and elongated nucleus in the *rad54Δ/rad54Δ *panel. C. Quantitation and examples of the nuclei morphology types seen in the *ard54Δ/rad54Δ *pseudohyphal cells. D. Quantitation and examples of the nuclei morphology in doublet cells in the WT and *rad54Δ/rad54Δ *cells.

To characterize the role of *RAD54 *and *RDH54 *in *Candida albicans*, deletion strains were made in the wildtype strain SC5314 using the *SAT1-FLP *technique described in [22]. Homozygous null transformants were obtained for both genes, indicating that neither was essential for growth in *Candida albicans*. Growth curves were performed in rich media (YPD) and revealed a growth defect in the *rad54Δ/rad54Δ *deletion mutant (Figure 1a). The *RAD54 *reconstruction strain did not have this defect, and grew as well as wildtype. The doubling times of each strain were calculated, and indicated that the heterozygous null mutants, the *rdh54Δ/rdh54Δ *mutant, and the *RAD54 *reconstruction strain all have doubling times comparable to SC5314, whereas the *rad54Δ/rad54Δ *strain had an increased doubling time (Figure 1b). Additionally, growth on solid media showed a decreased colony size in the *rad54Δ/rad54Δ *mutant when compared to the wildtype or reconstruction strains (Figure 2a). These results are similar to those obtained for other homologous recombination mutants in *Candida albicans*, as previously reported for *RAD52 *and *RAD51 *[23,24].

We attempted to construct the double mutant *rad54Δ/rad54Δ rdh54Δ/rdh54Δ *without success. The *RAD54/rad54Δ rdh54Δ/rdh54Δ *was fully viable and was identical to the single homozygous *rdh54Δ/rdh54Δ *mutant for all phenotypes assayed.

### Candida albicans RAD54 deletion causes altered cell and colony morphology

Growth of the *rad54Δ/rad54Δ *strain on YPD agar plates showed not only a decrease in colony size, but also a severe colony morphology defect. The colonies had a wrinkled appearance in contrast to the larger, smooth colonies of the parental strain and the *rdh54Δ/rdh54Δ *mutant. The heterozygous deletion mutants did not have altered colony morphology, and grew as smooth colonies as seen with the wildtype strain (data not shown). The altered colony morphology was rescued by reintroduction of *Candida albicans RAD54 *in the reconstruction strain (Figure 2a). Cell morphology of the *rad54Δ/rad54Δ *strain was also abnormal, with over 20% of cells exhibiting an elongated morphology reminiscent of pseudohyphae (Figure 2b). These cells did not appear to be true pseudohyphae, as they had a highly aberrant and variable morphology, similar to that seen in *Candida albicans *strains defective in cell cycle progression.

The numbers of cells with normal and abnormal morphology were quantitated and are shown in Table 1 and Figure 2c and 2d. When compared to wildtype, log phase cultures of the *rad54Δ/rad54Δ *strain had far fewer normal budding yeast cells, and a large increase in the number of cells exhibiting the abnormal morphology shown in Figure 2b. The elongated pseudohyphal cells displayed an aberrant nuclear morphology with a preponderance of the pseudohyphal cells having an elongated single DAPI staining body stuck in the neck between the two cell bodies (Figure 2c). Additional nuclear morphologies included apparent anucleate cells (two cells with only one nucleus), cells with a nucleus in each bud where one nucleus is elongated, and cells with multiple nuclei (Figure 2c). Regarding the pseudohyphal cells, in the single nucleate cells, 9/14 had an elongated single nucleus, and in the cells with two nuclei, 10/20 had one or two elongated nuclei.

**Table 1:** Log phase morphology of *Candida albicans *mutants

Strain	Unbudded	Budded	Abnormal/Pseudohyphae	Total
Wildtype	108	191	1	300
*rdh54Δ/RDH54*	111	187	2	300
*rdh54Δ/rdh54Δ*	78	221	1	300
*rad54Δ/RAD54*	71	227	1	300
*rad54Δ/rad54Δ-1*	92	143	65	300
*rad54Δ/RAD54*(+)	108	191	1	300

DAPI staining of cells also showed additional defects in chromosome segregation in the *rad54Δ/rad54Δ *strain. There was an increase in G2 doublet cells that have a single nucleus at the neck (Figure 2d). This morphology is suggestive of a DNA damage checkpoint arrest in *Saccharomyces cerevisiae *[25] and could apply to *Candida albicans *[26]. These phenotypes were not seen in the *rdh54Δ/rdh54Δ *strain, showing that these two genes have different roles in vivo. Additionally, neither the wildtype strain nor the *RAD54 *reintegration strain showed these aberrant nuclear morphologies.

### Sensitivity to DNA damage is increased in the Candida albicans rad54Δ/rad54Δ mutant

**Figure 3:**
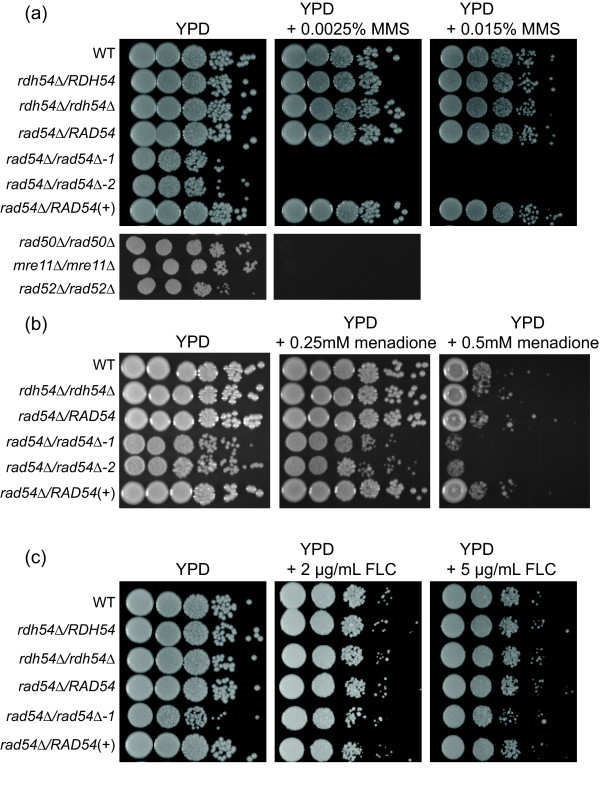
**MMS, menadione and FLC sensitivity of *rad54Δ/rad54Δ *and *rdh54Δ/rdh54Δ *strains**. Cells were grown as described in Materials and Methods, diluted and spotted onto plates with the indicated concentrations of MMS, menadione or FLC. The two *rad54Δ/rad54Δ *strains are independent transformants, designated as 1 and 2. Cells were photographed after 3 days growth at 30C. A. MMS sensitivity. B. Menadione sensitivity. C. FLC sensitivity.

In *Saccharomyces cerevisiae*, deletion of *RDH54 *and *RAD54 *leads to increased sensitivity to DNA damage. The *Saccharomyces cerevisiae *haploid *rad54Δ *is highly sensitive to methyl methanesulfonate (MMS) [19], but the *Saccharomyces cerevisiae RDH54 *gene does not appear to have as strong of a role in haploid cells, as deletion of *RDH54 *only increases MMS sensitivity in diploids at normal MMS concentrations [27]. To test the effect of deletion of *Candida albicans RAD54 *and *RDH54 *on MMS and menadione sensitivity, spot dilution assays were performed on YPD agar plates containing a range of MMS concentrations from 0.0025% to 0.02%, or menadione concentrations from 0.05 mM to 0.5 mM. Figure 3a shows that the *Candida albicans rad54Δ/rad54Δ *strain was exquisitely sensitive to MMS when compared to the wildtype strain, as sensitive as the previously reported *rad50Δ/rad50Δ, mre11Δ/mre11Δ *and *rad52Δ/rad52Δ *strains [12]. The *rad54Δ/rad54Δ *strain also had a moderate increase in sensitivity to oxidative damage from menadione (Figure 3b), similar to that reported for *rad50Δ/rad50Δ, mre11Δ/mre11Δ *and *rad52Δ/rad52Δ *strains [12]. The heterozygous deletion strains did not show increased MMS or menadione sensitivity, nor did the *rdh54Δ/rdh54Δ *homozygous deletion strain. Restoration of one *RAD54 *allele in the reconstruction strain restored the MMS and menadione sensitivity to wildtype levels.

### Susceptibility to antifungal drugs is not altered in the Candida albicans rad54Δ/rad54Δ and Candida albicans rdh54Δ/rdh54Δ mutants

Previous reports have linked genomic rearrangements with the development of FLC resistance in clinical isolates of *Candida albicans *[8,10]. Interestingly, defects in double strand break repair in laboratory generated *Candida albicans *mutants were previously shown to result in decreased susceptibility to FLC [12]. To test whether the homologous recombination proteins Rad54 and Rdh54 affect susceptibility to FLC, spot dilution assays were performed. The *rad54Δ/rad54Δ *mutant did not show any alteration in susceptibility to FLC, and this was corroborated by the E-test method. The *rdh54Δ/rdh54Δ *mutant had wildtype level of susceptibility (Figure 3c). The *rad54Δ/RAD54 *and *rdh54Δ/RDH54Δ *heterozygous mutants did not show increased susceptibility to FLC, and the *RAD54 *reconstruction strain also had FLC susceptibility similar to the wildtype strain (Figure 3). It appeared that better growing segregants arose at a higher frequency in the *rad54Δ/rad54Δ *mutant when plated on FLC-containing plates (Figure 3c). This would be consistent with a higher spontaneous mutation rated noted for *rad54Δ *and other homologous recombination mutants in *Saccharomyces cerevisiae *[28].

Susceptibility to other antifungals tested was also not altered for the mutants. Amphotericin B, 5-flucytosine and caspofungin were tested using the E-test method, and MIC values are shown in Table 2.

**Table 2:** Antifungal susceptibilities (MIC (μg/mL) of *Candida albicans *mutantsa

	Fluconazole	Amphotericin B	Caspsofungin	5-Flucystosine
Wildtype (SC5314)	1	0.64	0.094	2.0
*rdh54Δ/rdh54Δ*	0.5	0.64	0.064	2.0
*rad54Δ/RAD54*	1	0.64	0.094	2.0
*rad54Δ/rad54Δ-1*	0.5	0.64	0.064	2.0
*rad54Δ/RAD54*(+)	0.5	0.64	0.064	2.0

^a ^MICs were determined using standard E-test procedure on CAS plates. Values

were read after 48 hours of growth.

## Discussion

These studies address the role of *Candida albicans RAD54 *and *Candida albicans RDH54 *in DNA damage and azole sensitivity. This study shows that *Candida albicans RAD54 *and *Candida albicans RDH54 *are not essential genes. This is similar to deletion mutants of other homologous recombination genes such as *MRE11, RAD50 *and *RAD52 *[12,29]. Nonetheless, the *rad54Δ/rad54Δ *strain gave an aberrant colony morphology that suggested both a slower cell division time and checkpoint arrest to give lethal sectoring and a jagged colony edge. In contrast, the *rdh54Δ/rdh54Δ *strain grew with wildtype morphology and kinetics. Determination of cell cycle division times verified the slow growth phenotype of the *rad54Δ/rad54Δ *strain while the heterozygous and reconstructed *rad54Δ/RAD54 *strains grew with wildtype kinetics. Examination of individual cells corroborated the aberrant morphology and slower cell cycle time. The *rad54Δ/rad54Δ *strain accumulated cells with a pseudohyphal shape during log phase growth. DAPI staining of cells showed that nuclear division was aberrant, with the pseudohyphal cells often having one elongated DAPI-staining body. Additionally, the *rad54Δ/rad54Δ *strain had an excess of doublet (large-budded) cells with a single nucleus at the bud neck. This phenotype is suggestive of a checkpoint arrest and a defect in chromosome segregation. Interestingly, the aberrant morphology of the *rad54Δ/rad54Δ *strain also extends to growth on Spider medium. The *rad54Δ/rad54Δ *strain was defective in invasion of Spider agar when compared to the wildtype and reconstructed strains (data not shown), perhaps due to the altered morphology of the cells.

It is noted that this aberrant growth phenotype occurs in response to spontaneous damage. While diploid homozygous homologous recombination mutants in *Saccharomyces cerevisiae *grow slower than wildtype diploids, they do not show aberrant colony morphology. The *Saccharomyces cerevisiae rad54Δ/rad54Δ rdh54Δ/rdh54Δ *mutant shows an aberrant colony morphology similar to the *Candida albicans rad54Δ/rad54Δ *strain but is more extreme [14]. Attempts to make a *Candida albicans rad54Δ/rad54Δ rdh54Δ/rdh54Δ *double mutant were unsuccessful, suggesting that the double mutant may be lethal or grows extremely poorly. Homozygous deletions of *RAD54 *in chicken DT40 cells [30,31], mouse [32], and Drosophila [33] have resulted in sensitivity to ionizing radiation, MMS and crosslinking agents and defective meiosis, but have only a modest effect on cell growth, if discernible at all.

We assessed MMS sensitivity to determine the importance of the homologous recombination genes in DNA damage repair and found, similar to *Saccharomyces cerevisiae*, that *Candida albicans RAD54 *is extremely important for MMS damage repair and that *Candida albicans RDH54 *had no discernible role in MMS damage repair.

As FLC susceptibility has been linked to homologous recombination deficiency in *Candida albicans*, we determined the FLC susceptibility of the *rad54Δ/rad54Δ *and *rdh54Δ/rdh54Δ *strains. We also determined the susceptibility to several other antifungals by E-test analysis, to test whether genomic instability might result in altered susceptibility. The *rdh54Δ/rdh54Δ *and *rad54Δ/rad54Δ *strains did not exhibit any significant altered susceptibility to any of the antifungals tested. Additionally, the *rad54Δ/rad54Δ *and *rdh54Δ/rdh54Δ *strains showed no significant increase in FLC susceptibility above the reduced growth rate of the strain in the absence of FLC, suggesting that at least in the *rad54Δ/rad54Δ *strain, despite the obvious defects in nuclear segregation and cell division, these do not contribute to FLC resistance in the short term. It is possible that long term exposure to FLC might reveal a role for genomic instability and FLC resistance. It is also possible that the *rad54Δ/rad54Δ *mutant is buffered by the presence of the wild type *RDH54 *genes as regards FLC resistance, however the inability to recover the double mutant precludes a direct test of this hypothesis. We noted that strains segregated colonies of varying size on FLC and menadione plates. Such colonies could be candidates for segregants with mutations or genome rearrangements, but nature of the change and the rate of such segregants has not been determined.

## Conclusions

The results reported here support a role for homologous recombination genes *RAD54 *and *RDH54 *in DNA repair under nondamaging conditions. The nuclear morphology defects in the *rad54Δ/rad54Δ *mutants show that Rad54 performs an essential role during mitotic growth and that in its absence, cells arrest in G2, despite the presence of Rdh54. The viability of the single mutant *rad54Δ/rad54Δ *and the inability to construct the double mutant *rad54Δ/rad54Δ rdh54Δ/rdh54Δ *suggests that Rdh54 can partially compensate for Rad54 during mitotic growth, but that the two proteins have unique roles that contribute to cell viability.

## Methods

### Strains and growth conditions

*Candida albicans *wildtype strain SC5314 was used to construct all mutants created for this study. Deletion and replacement of *Candida albicans RAD54 *and *Candida albicans RDH54 *was done using the nourseothricin resistance marker *SAT1 *(generously provided by Dr. Joachim Morchauser) to create homozygous null mutants *Candida albicans rdh54Δ/rdh54Δ, Candida albicans rad54Δ/rad54Δ *and the reconstructed strain *Candida albicans rad54Δ/RAD54 *(+). The reconstructed strain *rad54Δ/RAD54 *(+) was made from one of the *rad54Δ/rad54Δ *strains. For routine growth, strains were maintained at 30°C on YPD (10 g Difco yeast extract, 20 g Bacto peptone, and 20 g dextrose per liter) with or without 200 μg/ml nourseothricin. Spider media was used for agar invasion assays, with a final pH of 7.2 (10 g nutrient broth, 10 g mannitol, 2 g K_2_PO_4 _and 25 g agar per liter).

### Plasmid construction

To create null mutants of *Candida albicans RAD54 *and *Candida albicans RDH54*, approximately 500 base pairs (bp) of sequence upstream and downstream of these ORFs was cloned on either side of the *SAT1*-*FLP *cassette in the vector pFS2A [22]. Fragments were PCR-amplified from SC5314 genomic DNA using the oligonucleotides listed in Table 3. The fragments were designed such that the entire coding sequence from ATG to the stop codon would be replaced by the *SAT1 *cassette. For both genes, the upstream fragment was cloned using the restriction enzymes *Apa*I and *Xho*I and the downstream fragment was cloned using *Not*I and *Sac*II. To create the *Candida albicans RAD54 *reconstruction vector, the entire coding region, including promoter and terminator sequence was cloned into the *Apa*I-*Xho*I site in the *Candida albicans RAD54 *deletion vector.

**Table 3:** List of oligonucleotides used in this study

Oligonucleotide name	5' - 3' sequence
CaRAD54upF	CAACGTAGGGCCCTCTAAAAATGTTGAAATTGG
CaRAD54upR	CAACGTACTCGAGGAGAATGGAAAGTACTGT
CaRAD54downF	CAACGTAGCGGCCGCTTTTAATATAAAACAATGTTG
CaRAD54downR	CAACGTACCGCGGAGGAATACTTGCAGTTGAC
CaRDH54upF	CAACGTAGGGCCCATGTACAAGATAAATTTG
CaRDH54upR	CAACGTACTCGAGCGCGTTGACAAAATTC
CaRDH54downF	CAACGTAGCGGCCGCCGCGTTTGACAAAATTC
CaRDH54downR	CAACGTACCGCGGCAAAAAGCACCAAAGTTG
CaRAD54compR	CAACGTACTCGAGAGGAATACTTGCAGTTGAC

Restriction site sequences are shown in bold

### Yeast transformation and screening

SC5314 was transformed with linearized (linearized with *Apa*I and *Sac*II) *Candida albicans RAD54 *or *Candida albicans RDH54 *deletion vectors using the standard lithium acetate method [34] with the following modifications. Heat shock at 42°C was carried out overnight, and cells were resuspended in YPD and allowed to grow for 4 hours at 30°C before plating on YPD containing 200 μg/mL cloNAT (Werner BioAgents, Jena, Germany). Recycling of the *SAT1 *marker was done by growing cells overnight in non-selective media (YPD) and plating onto YPD containing 25 μg/mL nourseothricin. Small colonies that had excised the marker were screened by PCR and used in a successive round of transformation. These tranformants were then screened by PCR for homozygous deletion of *Candida albicans RAD54 *and *Candida albicans RDH54*. To create the *Candida albicans RAD54 *reconstruction strain, recycling of the *SAT1 *marker was performed again and the reconstruction plasmid was introduced to the native locus by another round of transformation.

### Growth rate determination

Overnight YPD cultures from three independent colonies were used to inoculate 3 mL YPD at an OD_600 _of 0.05. Cultures were grown at 30°C with shaking. OD measurements were taken every hour for 9 hours to generate growth curves. Doubling times of each strain were calculated using time points within the logarithmic phase of growth. This assay was repeated three times, the mean and standard deviations for each strain is shown.

### Colony morphology and microscopic analysis

For assessment of colony morphology, cells were grown on YPD for 2 days at 30°C and single colonies were photographed. For colony invasion of agar, strains were streaked onto Spider agar plates (1% nutrient broth, 1% mannitol, 0.2% K_2_HPO_4 _and 20 g Bacto agar per liter) and incubated at 37°C for seven days and images were taken. For cell morphology, cells were grown in YPD to early log phase from YPD overnight cultures. Samples were taken, washed and resuspended in PBS buffer, and sonicated for 5 seconds at 30% amplitude in a Fisher Scientific 150T Series Sonic Dismembrator (Fisher Scientific, USA). Light microscopy was used to quantify numbers of single unbudded cells, budding cells, and cells with abnormal or pseudohyphal-like morphology. To assess nuclear integrity, cells were grown to early log phase and stained with DAPI according to a previously published protocol [35]. Overnight cultures were diluted to an OD_600 _of 0.05 in 5 mL of YPD and allowed to grow for 4 hours at 30°C. Samples were spun down, washed in 1 mL of 1X PBS, and fixed overnight at 4°C in 1 mL of 70% ethanol. Fixed cells were washed and treated for 2 hours in 55 mM HCl with 5 mg/mL pepsin at 37°C, then washed and resuspended in 1 mL of 1X PBS containing 2.5 μg/mL DAPI (Sigma-Aldrich, St. Louis, MO, USA). Cells were sonicated and visualized using a Zeiss Axio ImagerZ.1 microscope (Carl Zeiss Microimaging, Inc, Thornwood, NY, USA).

### DNA damage and antifungal drug sensitivities

To test the sensitivity of strains in this study to various agents, the agar spot dilution method was used. Overnight YPD cultures were diluted to an OD_600 _of 1.0 and serial ten-fold dilutions were made to 10^-6^. 2 μL volumes of each dilution were spotted onto YPD plates, and YPD plates containing FLC, MMS or menadione (Sigma-Aldrich, St. Louis, MO, USA) at the indicated concentrations. Plates were incubated for 48 hours at 30°C and images were taken. E-test analysis was performed for common antifungals, using overnight cultures diluted to an OD_600 _of 0.05 to spread a lawn on CAS plates (9.0 g casitone, 5.0 g yeast extract, 0.54 g KH_2_PO_4_, 3.34 g K_2_HPO_4_, 20. 0 g dextrose and 20.0 g agar per liter). E-test strips were placed on plates, which were incubated for 48 hours at 30°C. Two independent nulls of the *RAD54 *gene were tested. The MIC was read as the point at with the zone of inhibition intersected the E-test strip.

## List of abbreviations

FLC: fluconazole; LOH: loss of heterozygosity; MMS: methyl methanesulfonate; YPD: yeast extract-peptone-dextrose

## Competing interests

The authors declare that they have no competing interests.

## Authors' contributions

SJH carried out the mutant constructions, performed the DNA damage sensitivity tests and the DAPI microscopy and drafted the manuscript, XZ performed the dilution drop tests, CJP helped analyze the DAPI results and figure construction, TCW helped write the manuscript and in the interpretation of the mutant antifungal drug sensitivity tests. HLK and SJH conceived of the study. HLK designed some of the experiments and wrote the final manuscript. All authors have read and approved the final manuscript.
